# Impact of the COVID-19 Pandemic on Mental Health in Childhood and Adolescence: The Reality of a Portuguese School

**DOI:** 10.7759/cureus.29049

**Published:** 2022-09-11

**Authors:** Mariana Pedro, Marta Caldas, Jorge Penas, Bárbara Marques

**Affiliations:** 1 Pediatrics, Centro Hospitalar do Oeste, Unidade Caldas da Rainha, Caldas da Rainha, PRT

**Keywords:** covid-19 infection, covid-19 pandemic, mental wellness, depressive symptom, child and youth mental health

## Abstract

Background

The coronavirus disease 2019 (COVID-19) outbreak has led to social isolation, with the potential to increase depressive symptoms, even at the pediatric age. Before the COVID-19 pandemic, the rate of depressive symptoms in large youth cohorts was 12.9% worldwide.

Aims

This study aims to characterize the impact of the COVID-19 pandemic on the pediatric population’s mental health.

Materials and methods

This was an observational, descriptive, and cross-sectional study conducted through the use of a questionnaire, including the Children's Depression Inventory (CDI), between April 5 and May 5, 2021. The study was conducted on children and adolescents aged 7 to 17 years old in a school in the geographical area of ​​a Portuguese grade II hospital. Incomplete data were excluded. Data were statistically analyzed using the IBM SPSS® program (version 28; IBM Corp., Armonk, NY), considering statistical significance if p<0.05.

Results

A total of 228 children and adolescents were included; 113 were female (49.6%). The average age of the population was 12.2 years. Fifteen point four percent (15.4%) had depressive symptoms, of which 51,9% were female. Of the children and adolescents with depressive symptoms, 5.7% had a personal history of past COVID-19 infection and 42.9% had at least one family member with a history of past COVID-19 infection. Seventeen point one percent (17.1%) had at least one family member involved in pandemic-related work. Children and adolescents who were infected with COVID-19 had more depressive symptoms than noninfected children and adolescents (p=0.013). At the same time, children and adolescents, with at least one family member with a history of past COVID-19 infection, had more depressive symptoms than children and adolescents without a family history of past COVID-19 infection (p=0.004). Children and adolescents with a family member involved in pandemic-related work had more depressive symptoms than children and adolescents without any family member involved in pandemic-related work (p=0.004).

Conclusions

COVID-19 infection, whether personal or familiar, has an impact on mental health, even in the pediatric age, and it is imperative to know the consequences of emotional and mental changes in this population.

## Introduction

Coronavirus disease 2019 (COVID-19) was first detected in Wuhan, China, in December 2019; however, with globalization, there was a rapid spread of the disease. Thereby, on January 30, 2020, COVID-19 was declared a public health emergency, and almost two months later, on March 11, COVID-19 was declared a global pandemic [1]. Pandemics are infectious disease outbreaks that lead to a public health risk on a global scale.

Some preventive measures were applied in Portugal to prevent the spread of COVID-19, so on March 16, 2020, the Ministry of Health implemented disease containment measures such as school closures, social distancing, and home quarantine [2]. On May 6, 2020, schools were suspended in 177 countries, affecting over 1.3 billion learners worldwide [3]. However, in Portugal, the first lockdown was initiated previously, on March 16, 2020, and school in person just started again on September 14, 2020. The second lockdown was initiated on January 15, 2021, and ended on April 5, 2021. During lockdowns, schools worked as virtual learning classes. After a long period at home, in confinement, with strict measures, children and adolescents returned to school on April 5, 2021.

COVID-19 has both physical as well as mental sequelae. Previous pandemics and infection control measures had a direct effect on mental health [1]. One such example is the first outbreak of equine influenza in Australia, with 34% reporting high psychological distress, compared to levels of around 12% in the Australian general population [4].

Before the COVID-19 pandemic, rates of anxiety and depressive symptoms in global large youth cohorts were 11.6% and 12.9%, respectively [5]. Moreover, a Portuguese study described similar rates of depressive symptoms (11.2%) before the COVID-19 pandemic [6]. Additionally, the prevalence of depressive symptoms in youth increased during the COVID-19 pandemic in some cross-sectional [7,8] and longitudinal studies [9,10]. Those studies were related to ages below 18 years.

The aim of this study was to characterize the impact of infection control measures due to the COVID-19 pandemic on children and adolescents' mental health and to determine whether there are factors associated with an increase in depressive symptoms, such as age, local of residence, personal history of COVID-19 infection, family history of COVID-19 infection, the existence of a family member working on pandemic-related work, among others.

## Materials and methods

Inclusion and exclusion criteria

We conducted a questionnaire at an elementary and middle school with children and adolescents aged 7 to 17 years. For the selection of the schools, a simple random sampling was used and an equal number of classes was established for years of schooling. Questionnaires from children aged between 7 and 10 years were filled by their caretakers at home and surveys from children and adolescents aged between 10 and 17 years were filled by themselves inside the classroom.

A total of 228 participants completed the questionnaire from two different schools. However, 44 participants were excluded, as their questionnaire was incomplete and five participants were excluded, as their age did not fall in the age range of the study. Data collection took place from April 5, 2021, to May 5, 2021, after the lockdown restrictions were lifted to capture the situation of families, children, and adolescents during the most challenging pandemic time. 

Study instruments

The questionnaire consisted of two parts: (1) demographic characteristics of children and adolescents during the COVID-19 pandemic and (2) evaluate children for possible depression using the Children's Depression Inventory (CDI). The assessment of the existence of depressive symptoms was based on the CDI in Dias e Gonçalves's (1999) adaptation of the original CDI (Kovacs, 1985) [11,12].

The CDI is an instrument designed to measure signs of depression in children and adolescents aged 7 to 17 years (Cronbach's α value 0.97). The CDI is a 27-item scale that is self-reporting and symptom-based. The 27 items on the assessment are divided into five main factor domains: negative mood, interpersonal difficulties, ineffectiveness, anhedonia, and negative self-esteem. Each item consists of three statements ranked from 0 to 2 in order of increasing severity. Children select the one that best describes their symptoms in the past two weeks. Item scores are summed for a total depression score, ranging from 0 to 54. The discriminative index of CDI for depressive symptoms is 16 for the ages between 8 to 12 years and 20 for the ages between 13 to 17 years [12].

The children's data that was analyzed included gender, age, children, and parents' educational level, place of residence (city or village), number of siblings, involvement of a family member in pandemic-related work, personal history of COVID-19 infection, family history of COVID-19 infection, and personal rate of concern about the COVID-19 pandemic.

Statistical models

Chi-squared tests were used to compare children and adolescents with or without depressive symptoms with age, personal history of COVID-19 infection, family member with a history of COVID-19 infection, and family member working on pandemic-related work. Chi-squared tests were also used to compare the rate of concern about the COVID-19 pandemic between children and adolescents with or without depressive symptoms, personal history of COVID-19 infection, a family member with a history of COVID-19 infection, and a family member working on pandemic-related work. A p-value of < 0.05 was considered statistically significant in all analyses. Analyses were performed using SPSS 26.0 (IBM Corp., Armonk, NY).

Ethics approval and informed consent

Ethical approval was obtained from the Centro Hospitalar do Oeste Ethics Committee. We obtained written informed consent from the caretakers or guardians on behalf of the students.

## Results

The study included 228 children and adolescents, aged between 7 and 17 years. One-hundred thirteen (113) were female (49.6%) and the average age was 12.2 years. One-hundred fifty (150) respondents (65.8%) lived in a rural setting. One-hundred five (125) respondents (54.8%) had only one sibling. Most parents had an educational level below the high school level. Eleven point four percent (11.4%) of children and adolescents in this study failed at least one year at school (n=26) (Table 1).

**Table 1:** Demographic characteristics of the sample

Demographic variable	Subcategory	n	Percentage (%)
Gender	Female	114	50.0
	Male	110	48.2
	Prefer not to say	4	1.8
Age	7	12	5.3
	8	17	7.5
	9	18	7.9
	10	17	7.5
	11	30	13.2
	12	32	14.0
	13	25	11.0
	14	45	19.7
	15	26	11.4
	16	5	2.2
	17	1	0.4
Children and adolescents' school year	2^nd^	14	6.1
	3^rd^	18	7.9
	4^th^	20	8.8
	5^th^	20	8.8
	6^th^	48	21.1
	7^th^	16	7.0
	8^th^	42	18.4
	9^th^	50	21.9
Local of residence	Rural	150	65.8
	City	78	34.2
Number of siblings	0	51	22.4
	1	125	54.8
	2	41	18.0
	3	6	2.6
	>4	5	2.2
Mother’s educational level	Elementary school	63	27.6
	High school	78	34.2
	University degree	57	25.0
	Master's degree	21	9.2
	PhD degree	9	4.0
Father’s educational level	Elementary school	105	46.1
	High school	61	26.8
	University degree	38	16.7
	Master degree	14	6.1
	PhD degree	10	4.4
Children and adolescents who fail at least one year at school	Yes	26	11.4
	No	202	88.6
Family member working on pandemic-related work	Yes	41	18.0
	No	187	82.0
Personal history of COVID-19 infection	Yes	11	4.8
	No	217	95.2
Family history of COVID-19 infection	Yes	93	40.8
	No	135	59.2

Five point seven percent (5.7%) had a personal history of COVID-19 infection and 42.9% had at least one family member with a history of COVID-19 infection. Of all the participants, 17.1% had at least one family member working on pandemic-related work.

Regarding the COVID-19 pandemic’s rate concern, 67% of children and adolescents reported that were “worried” or “very worried” about the COVID-19 pandemic (n=154). Children and adolescents with at least one family member with a COVID-19 past infection showed a greater rate of concern, reporting that they were “very worried” and “worried” (75.3%), than children and adolescents without a family member's history of COVID-19 past infection (62.2%) (p=0.0387). Curiously, the same was not observed in children and adolescents with a personal history of COVID-19 past infection or with at least one family member working on pandemic-related work. Children and adolescents living in a village showed a greater rate of concern, as “very worried” and “worried” (72.7%), than children and adolescents living in a city (57.7%) (p=0.022). It is also interesting to share that children and adolescents without depressive symptoms showed a greater degree of concern, as “very worried” and “worried” (70.5%), than the group with depressive symptoms (51.4%) (p=0,0269). We summarize these findings in Table 2.

**Table 2:** Summarizing the degree of concern and other variables

	Degree of concern - “very worried” and “worried” (%)	Degree of concern - “more or less” and “not worried” (%)	p
Depressive symptoms	51.4	48.6	0.0269
No depressive symptoms	70.5	29.5	
Family member with a history of COVID-19 infection	75.3	24.7	0.0387
No family member with a history of COVID-19 infection	62.2	37.8	
Personal history of COVID-19 infection	66.7	33.3	0.798
No personal history of COVID-19 infection	70.7	29.3	
Family member working on pandemic-related work	78.0	22.0	0.1127
No family member working on pandemic-related work	65.2	34.8	
Living in a village	72.7	27.3	0.022
Living in a city	57.7	42.3	

Fifteen point four percent (15.4%) of participants had depressive symptoms after CDI application (Tables 3-4).

**Table 3:** Results of Children’s Depression Inventory (CDI)

Question	n	Percentage (%)
I get sad from time to time	195	85.5
I get sad often	29	12.7
I am always sad	4	1.8
For me, everything will work out	123	53.9
I am not sure if things will work out for me	92	40.4
Nothing is going to work out for me	13	5.7
I do well in most things	144	63.1
I do wrong most things	80	35.1
I do everything wrong	4	1.8
I have fun with many things	140	61.4
I have fun with some things	86	37.7
Nothing is fun for me	2	0.9
I am mean from time to time	206	90.4
I am often mean	19	8.3
I am always mean	3	1.3
From time to time, I think that bad thing will happen to me	107	46.9
I fear that bad things happen	93	40.8
I am sure that terrible things will happen to me	28	12.3
I like myself	191	83.8
I do not like myself	30	13.1
I hate myself	7	3.1
Normally I do not feel guilty for the bad things that happen	162	71.1
Many bad things that happen are my fault	57	25.0
Everything bad that happens is my fault	9	3.9
I do not think about killing myself	183	80.3
I think about killing myself, but I would not do	43	18.8
I want to kill myself	2	0.9
I feel like crying from time to time	180	78.9
I often feel like crying	40	17.6
I feel like crying every day	8	3.5
I get bored from time to time	163	71.5
Often I get bored	50	21.9
I am always bored	15	6.6
I like being with people	195	85.5
Often, I do not like being with people	28	12.3
I do not like being with people	5	2.2
I make decisions easily	113	49.6
It is hard for me to make decisions	110	48.2
I never make decisions	5	2.2
I am good looking	125	54.8
My appearance has some negative aspects	93	40.8
I am ugly	10	4.4
It is not difficult to do homework	94	41.2
I often have to work hard to do homework	81	35.5
I always have to work hard to do homework	53	23.3
I sleep well at night	145	63.6
I have trouble to sleep some nights	72	31.6
I always have trouble to sleep at night	11	4.8
I get tired from time to time	146	64.0
I often get tired	58	25.4
I am always tired	24	10.6
I always eat well	164	71.9
Often I do not want to eat	48	21.1
Most days I don’t feel like eating	16	7.0
I am always worried with my health	48	21.1
I care a lot about my health	159	69.7
I do not care about my health	21	9.2
I do not feel alone	169	74.1
I often feel alone	56	24.6
I always feel alone	3	1.3
I often have fun at school	163	71.5
I have fun at school from time to time	60	26.3
I never have fun at school	5	2.2
I have lots of friends	167	73.2
I have lots of friends, but I would like to have more	57	25.0
I have no friends	4	1.8
The grades at school are good	115	50.4
The grades at school used to be better	98	43.0
I’m very bad in subjects that I used to be very good at	15	6.6
I am as good as other children	98	43.0
If I want, I can be as good as other children	97	42.5
I can not be as good as other children	33	14.5
I’m sure that I am loved by someone	172	75.4
I’m not sure if anyone loves me	53	23.3
Nobody really likes me	3	1.3
I always do what I’m told	156	68.4
I often do not do what I am told	69	30.3
I never do what I am told	3	1.3
I get along well with other	213	93.4
I am often involved in discussions	14	6.2
I am always involved in discussions	1	0.4

**Table 4:** Description of the Children’s Depression Inventory score results

	Discriminative index of CDI for depressive symptoms	Minimum score	Maximum score	Mean score	Median score	Mode score
Aged 8-12	CDI ≥ 16	0	35	10.44	9	6
	n = 17					
Aged 13-17	CDI ≥ 20	1	35	12.16	10	10
	n = 18					

The average age of children with depressive symptoms were 12.83 years. Remembering that the discriminative index of CDI for depressive symptoms is 16 (CDI score) for the ages between 8 and 12 years and 20 (CDI score) for the ages between 13 and 17 years. The minimum age of depressive symptoms was 8 years and the maximum age was 17 years. Additionally, children with depressive symptoms were older than children without depressive symptoms (p=0.002). There is no statistically significant difference between children with depressive symptoms who lived in the city or in the village.

Children and adolescents with a personal history of a past COVID-19 infection presented more depressive symptoms (18.2%) than children and adolescents without a personal history of a past COVID-19 infection (15.2%) (p=0.013).

Children and adolescents with at least one family member with a history of a past COVID-19 infection presented more depressive symptoms (16.1%) than children and adolescents without any family history of a past COVID-19 infection (14.8%) (p=0.004).

Children and adolescents with at least one family member working on pandemic-related work had more depressive symptoms than children and adolescents without any family member working on pandemic-related work (p=0.004).

## Discussion

This study conducted in Portugal on children ages 7 to 17 years old found that 15.4% of these children exhibited signs of depression using the CDI scale. Schools were closed for almost nine months due to the isolation protocols for the COVID-19 pandemic from March 16, 2020, to September 14, 2020, and again from January 15, 2021, to April 5, 2021. This survey was conducted as the children returned to school once the infection control restrictions eased allowing the school to reopen. Our results suggest an increase in psychological distress during the pandemic, as a Portuguese study described a rate of pre-pandemic depressive symptoms of 11.2% in this age group [6], suffering an increase to 15.4% with the COVID-19 pandemic. To our knowledge, this is one of the first studies that show an association between depressive symptoms and the COVID-19 pandemic in a sample of children and adolescents in Portugal [13].

The present study aimed to measure the psychological impact of COVID-19 restrictive measures and school closure on children and adolescents during one of the most unique periods in recent history, in which children and adolescents did not go to school for weeks and had limited contact with friends and family. The results showed that a significant percentage of children and adolescents had more depressive symptoms than before the COVID-19 pandemic. This finding is consistent with previous studies of childhood and adolescent mental illness before the COVID-19 pandemic, which showed lower rates of clinically depressive symptoms [5,6,14,15].

Current findings suggest that closure measures have worsened rates of depressive symptoms in children and adolescents [16,17]. In addition, schools are often the primary site for mental health services. Eighty percent (80%) of children rely on school-based services to improve their mental health. For many children, these services had been unavailable due to school closures, and the schools included in this study also did not have these services available [5].

In a meta-analysis, the prevalence rates of depressive symptoms were higher as child age increased, and we reported the same in our study [5]. 

Furthermore, our study showed no difference between children and adolescents living in a village or a city regarding depressive symptoms, however, there is a difference in the rate of concern because children and adolescents living in a village reported a higher degree of concern (72.7%) as compared with children and adolescents living in a city (57.7%).

Nevertheless, other studies found that a harmonious social environment, like a rural environment, is more positively associated with better mental health [18]. In our study, children and adolescents living in a village reported a greater rate of concern, but this is difficult to clarify in other studies.

As the results showed, children and adolescents with a personal or familial history of a past COVID-19 infection had more depressive symptoms, as well as if there was any family member working on pandemic-related work. However, to our knowledge, there are no other studies in the literature that support this; therefore, it would be important to extend this study to other schools in Portugal, in order to justify the measures to be adopted.

Hence, it would be important to establish some protective measures to improve this situation. Additional resources and training would likely be needed to prepare teachers to support children with low moods and increase their awareness of referral pathways for professional support, as previous studies also suggested [19].

It would be important to increase the number of psychologists available in schools, in order to follow children and adolescents with warning signs. Children and adolescents with a personal or familial history of a past COVID-19 infection should be flagged at school and early psychological work should be initiated, in order to prevent the increase of depressive symptoms. Additionally, it would be important to talk with parents of children and adolescents who have family members working on pandemic-related work, in order to recognize possible signs of depression.

An important conclusion from the current findings is that the potential link between disease containment measures and children's mental health may be included in the decision-making process of policymakers.

It would have been interesting if we had assessed the familial functionality and the presence of depressive symptoms in the parents of children and adolescents, as other studies showed that 28.3% of children with mental health problems lived in a family that reported problems with family functioning, compared with 11.7% of children without mental health problems [20]. The same study, curiously, found that children and adolescents whose parents had a mental illness were less likely to receive regular support at school.

There are some limitations in our study. First, is not possible to obtain a causal explanation of the results due to the nature of this study. Second, the small sample size reduces the statistical power and precision of estimates. The current study does not have the statistical power to detect small but clinically significant changes. Third, because it is a random sample, the proportion of responders is relatively small compared with the size of the overall cohort. Finally, caution should be exercised in generalizing the results to other populations, because we surveyed only some schools of a city in the Lisbon region.

## Conclusions

We reported evidence of the negative association between disease containment measures and children’s mental health. In particular, we observed an increase in the rating of depression in Portuguese children and adolescents versus previous Portuguese studies present in the literature, from 11.2% to 15.4%. We also observed an increased rating of depression in children and adolescents with a personal or family history of a past COVID-19 infection, as well as if any family member had pandemic-related work. Children and adolescents with a personal and family history of a past COVID-19 infection presented more depressive symptoms, 18.2% and 16.1%, respectively.

It is advisable to conduct further studies in other schools and communities with detailed reports and provide a clear assessment of the mental health of children and adolescents both before and after the COVID-19 pandemic. Thus, it is important to understand the consequences of emotional and mental changes in this population, in order to implement measures to reduce the impact of the COVID-19 pandemic on childhood mental health.
